# Metabolome-Informed Microbiome Analysis Refines Metadata Classifications and Reveals Unexpected Medication Transfer in Captive Cheetahs

**DOI:** 10.1128/mSystems.00635-19

**Published:** 2020-03-10

**Authors:** Julia M. Gauglitz, James T. Morton, Anupriya Tripathi, Shalisa Hansen, Michele Gaffney, Carolina Carpenter, Kelly C. Weldon, Riya Shah, Amy Parampil, Andrea L. Fidgett, Austin D. Swafford, Rob Knight, Pieter C. Dorrestein

**Affiliations:** a Collaborative Mass Spectrometry Innovation Center, Skaggs School of Pharmacy and Pharmaceutical Sciences, University of California, San Diego, La Jolla, California, USA; b Skaggs School of Pharmacy and Pharmaceutical Sciences, University of California, San Diego, La Jolla, California, USA; c Center for Microbiome Innovation, University of California, San Diego, La Jolla, California, USA; d Center for Computational Biology, Flatiron Institute, New York, New York, USA; e Division of Biological Sciences, University of California, San Diego, La Jolla, California, USA; f Nutritional Services, San Diego Zoo Global, San Diego, California, USA; g Department of Pediatrics, School of Medicine, University of California, San Diego, La Jolla, California, USA; h Department of Computer Science & Engineering, University of California, San Diego, La Jolla, California, USA; i Department of Bioengineering, University of California, San Diego, La Jolla, California, USA; Teagasc Food Research Centre

**Keywords:** metabolome, metagenome, microbiome, cheetah, medication, antibiotics, metadata, *Acinonyx jubatus*

## Abstract

Even high-quality collection and reporting of study metadata in microbiome studies can lead to various forms of inadvertently missing or mischaracterized information that can alter the interpretation or outcome of the studies, especially with nonmodel organisms. Metabolomic profiling of fecal microbiome samples can provide empirical insight into unanticipated confounding factors that are not possible to obtain even from detailed care records. We illustrate this point using data from cheetahs from the San Diego Zoo Safari Park. The metabolomic characterization indicated that one cheetah had to be moved from the non-antibiotic-exposed group to the antibiotic-exposed group. The detection of the antibiotic in this second cheetah was likely due to grooming interactions with the cheetah that was administered antibiotics. Similarly, because transit time for stool is variable, fecal samples within the first few days of antibiotic prescription do not all contain detected antibiotics, and the microbiome is not yet affected. These insights significantly altered the way the samples were grouped for analysis (antibiotic versus no antibiotic) and the subsequent understanding of the effect of the antibiotics on the cheetah microbiome. Metabolomics also revealed information about numerous other medications and provided unexpected dietary insights that in turn improved our understanding of the molecular patterns on the impact on the community microbial structure. These results suggest that untargeted metabolomic data provide empirical evidence to correct records and aid in the monitoring of the health of nonmodel organisms in captivity, although we also expect that these methods may be appropriate for other social animals, such as cats.

**IMPORTANCE** Metabolome-informed analyses can enhance omics studies by enabling the correct partitioning of samples by identifying hidden confounders inadvertently misrepresented or omitted from carefully curated metadata. We demonstrate here the utility of metabolomics in a study characterizing the microbiome associated with liver disease in cheetahs. Metabolome-informed reinterpretation of metagenome and metabolome profiles factored in an unexpected transfer of antibiotics, preventing misinterpretation of the data. Our work suggests that untargeted metabolomics can be used to verify, augment, and correct sample metadata to support improved grouping of sample data for microbiome analyses, here for nonmodel organisms in captivity. However, the techniques also suggest a path forward for correcting clinical information in microbiome studies more broadly to enable higher-precision analyses.

## Abstract (precis)

Metabolome-informed analyses can enhance omics studies by enabling the correct partitioning of samples by identifying hidden confounders inadvertently misrepresented or omitted from carefully curated metadata. We demonstrate here the utility of metabolomics in a study characterizing the microbiome associated with liver disease in cheetahs. Metabolome-informed reinterpretation of metagenome and metabolome profiles factored in an unexpected transfer of antibiotics, preventing misinterpretation of the data. Our work suggests that untargeted metabolomics can be used to verify, augment, and correct sample metadata to support improved grouping of sample data for microbiome analyses, here for nonmodel organisms in captivity. However, the techniques also suggest a path forward for correcting clinical information in microbiome studies more broadly to enable higher-precision analyses.

## INTRODUCTION

The microbiome is accepted as a critical aspect of organismal health, with much attention being focused on the gut microbiome. This environment is variable, in part defined by an irregular flow of inputs, including diet and medications such as antibiotics, that impact and shape the microbial community (1–7). Common ways to examine this variability are to profile microbial community structure and functional capacity, and less frequently functional activity and output, including metabolite signatures. Correct interpretation of these profiles relies on the detailed, relevant metadata that form the foundation for all analyses and applications of the data.

A mismatch between collected metadata and variables of interest can result from a multitude of reasons, with reporting errors and biases, omission of categories in the metadata based on original study design, unexpected exposures, and poorly structured or inconsistent descriptors among the most common. The approach of some large human cohorts, such as the American Gut Project (8), has been to capture an extensive array of information using controlled vocabulary and values to maximize the likelihood of having relevant metadata. However, cohorts that rely on self-reported information and self-initiative to complete run the risk of obtaining erroneous, incomplete, and variable amounts of metadata for each sample. Social pressures or fear of repercussions may further prevent the disclosure of illicit or sensitive information such as drug use, sexually transmitted diseases, poor hygiene or diet, etc., and the length of time between a sampling event and collection of information about diet, medication use, or health-related events can further decrease accuracy (9). For example, metabolite analysis detected the presence of antibiotics in fecal samples from individuals who reported not having taken antibiotics in the past 6 months or more (8). Furthermore, a central issue across microbiome studies is that it is very challenging to capture additional participant information after a study has been completed, either due to the self-reported nature, unresponsive subjects, or simply the passage of time.

We propose that metabolite-informed microbiome analyses, where the small-molecule composition of a sample, readily detected using a liquid chromatography-tandem mass spectrometry (LC-MS/MS) workflow, can be used to generate empirically determined metadata assignments for compounds such as medications, including antibiotics or painkillers, and personal care products, such as sunscreen. In particular, the use of antibiotics in a clinical setting is common, impacting organisms from livestock, domestic animals, captive wildlife, to humans of every age. Antibiotics have a strong documented impact on the gut microbial community (1–5), often resulting in large decreases in alpha diversity. These changes in alpha diversity have the potential to alter microbial community structure and the gut metabolome. Antibiotics have also been shown to influence the gut microbial communities of members of a household where one individual is taking antibiotics (10). The route of impact was, however, not clear, leaving open the possibility of a shift in the microbiome that is microbially or chemically mediated, possibly through transfer of the drug itself between individuals.

Similar to how domestic animals and human patients are treated in a clinical setting, animals in managed care such as cheetahs (Acinonyx jubatus) have interventions only when deemed medically necessary. Based on the individual animal health history, each animal has a unique combination of housing location, diet, medication use, and environmental exposure. Cheetahs in captivity suffer from higher rates of veno-occlusive disease and gastrointestinal distress than their wild counterparts (11), leading to multiple treatment interventions, such as changes in diet and medications. However, unlike human subjects, zoo animals do not self-report information, and detailed records of food consumption, medication use, health parameters, housing, and behavior are recorded by keepers and trainers to capture these interventions. These detailed metadata and controlled conditions provide an ideal setting for examining the complementarity of a metabolome-informed approach to microbiome analyses.

**FIG 1:**
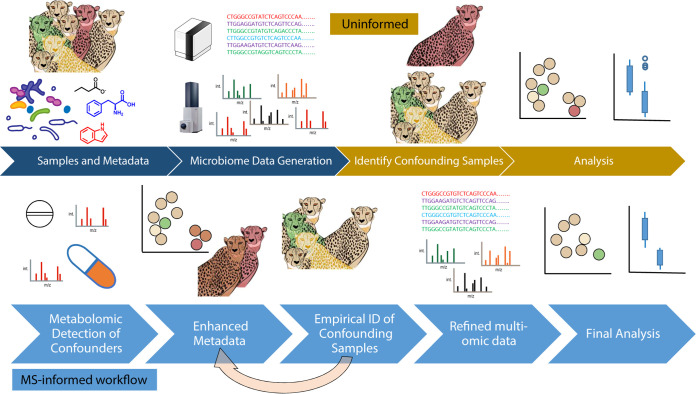
Analysis workflow for metabolome-informed microbiome analyses. All analyses begin with the collection of samples and metadata (information about the samples such as time of collection, subject name or number, etc.), followed by microbiome data generation, such as sequence data and untargeted metabolomics (dark blue). A traditional, or uninformed, analysis will then identify confounders from the metadata and individually parse the data sets based on reported metadata variables and synthesize results from the data sets in the last analysis step. In contrast, a metabolome-informed analysis, as presented in this study, will empirically determine whether there are confounders or additional information identified by metabolomic analysis (Fig. 2), which in this study identified antibiotics in samples where none were reported. The MS data empirically support creation of an MS-informed metadata column, which is applied to each data set collected (metagenome and metabolome in this analysis) (Fig. 3). This cycle can be iterated multiple times based on potential confounders and information obtained through the MS analysis. In this case study, a further iteration filtered the metabolite feature table itself, as medication-derived metabolites, given only to ill animals, dominated the differences observed (Fig. 4). MS-informed metadata allow the data themselves to help guide the analysis and facilitates communication between the data sets.

Here, we present a workflow (Fig. 1) for generating study-specific metabolome-informed metadata categories, using cheetahs as a case study, and highlight the value of the approach for generating empirical metadata and reinterpreting the data. Finally, we discuss broader applications of using empirical evidence to correct sample categorization and records and provide concrete examples where this technique is anticipated to have the greatest impact, suggesting areas where metabolomic data should be routinely collected.

## RESULTS

We collected and analyzed paired shallow shotgun metagenomic sequence data and untargeted LC-MS/MS data from fecal samples from seven cheetahs housed at the San Diego Zoo Safari Park in 2018 over the course of 1 month (Table 1). Shallow shotgun metagenomic sequencing (12) provides a snapshot of the microbial community structure at each time point and can provide insight into functional potential (e.g., the potential exchange and transformation of metabolites between the environment, microbes, and host). Untargeted LC-MS/MS metabolomic analysis aims to detect all molecules in a sample without any knowledge of the molecular constituents *a priori*, thereby assessing the chemistry of individuals as well as differences between individuals or populations. Broad untargeted metabolomics is able to identify compounds that corroborate or challenge metadata assignments and can thus be inspected in a concerted fashion for accuracy based on individual study design (Fig. 1) and therefore provide valuable empirical evidence to enhance the accuracy of our analyses. Combined, these data provide a window into the community structure as well as the inputs that are entering the gut microbial environment, including host, diet, and medication-related compounds.

**TABLE 1:** Overview of cheetah cohort and selected variables from the metadata^*a*^

Animal name	Accession no.	Sex^*b*^	DOB^*c*^	Age	Liver health	Antibiotic use
Amara	609025	F	18-Feb-09	9 yr 5 mo	Mild disease	No
Bahati	614426	F	1-Sep-14	3 yr 11 mo	Normal	No
Johari	609201	F		9 yr 1 mo	Severe disease	No
Isoka	615391	M	1-Sep-14	3 yr 11 mo	Normal	Yes
Kiburi	610376	M	15-Nov-10	7 yr 8 mo	Normal	No
Okubi	615390	M	1-Sep-14	3 yr 11 mo	Normal	No
Ruuxa	614198	M	3-May-14	4 yr 2 mo	Normal	No

a The analyses focus on seven cheetahs at Wildlife Discoveries at the San Diego Zoo Safari Park. Shotgun metagenomic and metabolomic data are available under Qiita ID 11872 and GNPS ID MSV000082969. Cheetahs maintained the same diet during the 30-day sampling time course.

b F, female; M, male.

c DOB, date of birth given in the form day-month-yr (months [February, September, and November] and years [2009, 2010, and 2014] are abbreviated).

### Discovery of inconsistency between reported and detected MS data.

**FIG 2:**
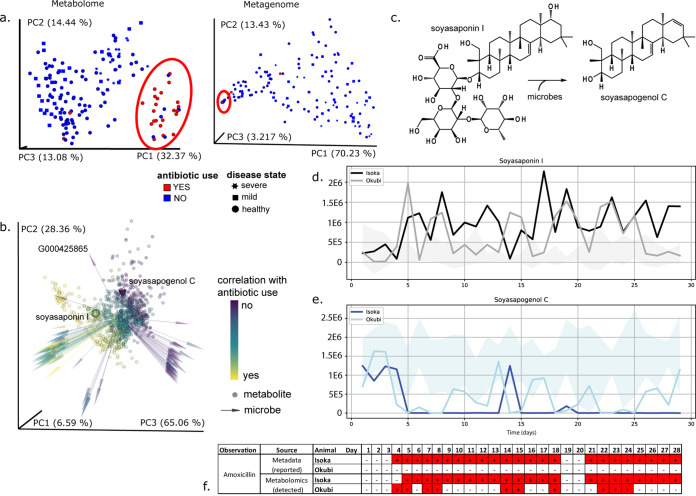
Antibiotics are a major driver of metabolome and metagenome beta diversity, and metabolome analysis reveals unexpected antibiotic transfer, corroborated by differential microbially mediated food metabolism. (a) Principal-coordinate analysis (weighted UniFrac) for MS feature abundance (left) and shotgun sequence data (right) for fecal samples from seven cheetahs housed at Wildlife Discoveries. Data points are colored by reported antibiotic use based on initial metadata (blue for no; red for yes). The shape of the symbol designates the diagnosed disease state, with regard to cheetah liver necrosis syndrome (CLNS). The metabolome is shown on the left, and the metagenome is shown on the right. The distance metric is weighted UniFrac. The red ovals highlight the region containing the samples from animals with antibiotic use. (b) Microbe-metabolite cooccurrence analysis. The large cone represents soyasapogenol C, and the large sphere represents soyasaponin I. In the biplot of cheetah data points, spheres are metabolites, and arrows are microbes. Both metabolites and microbes are colored by the same scale based on differential abundance analysis: yellow is associated with antibiotics; purple is not associated with antibiotics. The top 100 species from differential abundance analysis are displayed in the plot; all metabolites are shown. Web of Life genome ID: G000425865; NCBI taxonomy: k__Bacteria; p__Firmicutes; c_Bacilli; o_Lactobacillales; f__Carnobacteriaceae; g__*Lacticigenium*; s__*Lacticigenium naphtae*. (c) Microbially mediated conversion of soyasaponin I to soyasapogenol C. (d) Soyasaponin I abundance over time for Isoka (black), Okubi (gray) and the range of values for the five cheetahs (Amara, Bahati, Johari, Kiburi, and Ruuxa) with no detected amoxicillin (shaded gray). (e) Soyasapogenol C abundance over time, as plotted in panel d. Soyasapogenol C is consistently more abundant in feces than soyasaponin I. (f) Reported amoxicillin administration for Isoka by sampling day (reported), compared with detection of amoxicillin in fecal samples from Isoka and Okubi (detected). Antibiotic prescription or detection are highlighted in red. Days line up with the plots of feature abundance for soyasaponin I and soyasapogenol C. Feature data: soyasaponin I (*m/z* 441.3731, RT 7.3547 min, row ID 209, annotation network number *n*/*z*, correlation group ID 79); soyasapogenol C (*m/z* 943.5270, RT 5.3381 min, row ID 578, annotation network number 60 correlation group ID 58).

Initial inspection of the metabolome and metagenome identified antibiotic use as a main driver of differences between samples, as observed by principal-coordinate analysis (PCoA) (Fig. 2a and b), with the first principal axis (PC1) explaining 32.37% of the variance in the metabolome and 70.23% of the variance in the microbiome. The between sample distances from PCoA clearly differentiated the majority of the fecal samples belonging to the male cheetah Isoka, who was treated for gastritis with medication, including two 14-day courses of the antibiotic amoxicillin. Interestingly, a fraction of samples reported in the metadata as “no antibiotic use” cluster together with the “antibiotic use” samples, indicating a molecular similarity as well as microbial similarity.

We traced the origin of these “no antibiotic use” samples to one individual, the male cheetah Okubi (Fig. 2a and b, blue within the red oval) who did not receive any medications during the sampling period. Okubi was cohoused with his antibiotic-treated male sibling Isoka, while the other five cheetahs each had their own separate enclosures at the Wildlife Discoveries facility at the San Diego Zoo Safari Park. As cohousing presented a risk for misidentification of the source animal for fecal samples, medications were individually orally administered and glitter was added to Isoka’s diet (prescribed antibiotic [Rx antibiotic]), in order to verify which stool sample originated from which animal. Unlike captive rodents and some domestic animals, cheetahs are not coprophagic, i.e., do not eat each other’s stool. Furthermore, they cover and avoid their feces, which are removed daily by keepers, thus limiting the potential routes of molecular sharing. Instead, we hypothesize that grooming and other social interactions (13) could have led to direct medication carryover.

We tested this hypothesis by examining whether any of the antibiotics Isoka received were detected in the metabolomic data of the fecal samples from Okubi. The hydrolyzed form of amoxicillin was detected by library identification in samples from both Isoka (Rx antibiotic) and Okubi (no Rx) using the web-based global natural products (GNPS) analysis platform (https://gnps.ucsd.edu) (14), which provides Metabolomics Standards Initiative level II or III identifications (15). Moreover, this antibiotic was not detected in any samples from the other cheetahs. During the month-long time course, Isoka was on prescription antibiotics for 24 days (out of 29 total samples), and the amoxicillin derivative was detected in 23 samples from Isoka and intermittently in 10 samples from Okubi, which also group together based on PCoA (squares; Fig. 2f).

Using a multiomic microbe-metabolite cooccurrence analysis, we observed a strong trend with antibiotic use across both data sets, with highly correlated microbes and metabolites also cooccurring (Fig. 2b). Untargeted metabolomics identified members of the molecular family of plant products, soyasaponins, and their degradation products (such as that in Fig. 2c) in the stools of cheetahs consuming Nebraska Brand Special Beef Feline Diet. These ingredients, not initially part of the metadata collection, were confirmed by the manufacturer ingredient list of the dietary product (http://www.nebraskabrand.com/docs/beefsheet2019pdf.pdf).

One class of correlated molecules includes the soyasaponins, where we identified both precursor (soyasaponin I) and metabolite (soyasapogenol C). Previously, microbial fermentation has been implicated with the conversion of these compounds to their aglycone metabolites (16). These results support our findings, as the microbially derived metabolite soyasapogenol C is correlated with no antibiotic use, while the precursor is correlated with antibiotic use (Fig. 2b). In addition to these observed changes in the metabolome following antibiotic exposure, the presence of soyasapogenol C has a high cooccurrence probability with *Firmicutes* and *Proteobacteria*, with G000425865 among the most differential microbes in the analysis (see Table S1 in the supplemental material), indicating that changes in microbiota due to transfer of metabolites between individuals may be driving differences in metabolic function within the gastrointestinal tract.

10.1128/mSystems.00635-19.1 TABLE S1 Top 20 species ranked based on cooccurrence probability with the metabolite soyasaponin I. Genome ID assignments were obtained from the Web of Life (https://biocore.github.io/wol). Download Table S1, CSV file, 0.01 MB. Copyright © 2020 Gauglitz et al. 2020 Gauglitz et al. This content is distributed under the terms of the Creative Commons Attribution 4.0 International license.

### Metabolome-informed metadata grouping supported by altered microbial metabolism of diet components.

Further longitudinal assessment of soyasaponin I and soyasapogenol C (Fig. 2d and e) show differential metabolism of soy carbohydrates and mirror the medication schedule for Isoka (Rx antibiotic; Fig. 2f), and this empirical evidence supports restructuring of the metadata from antibiotic reported to detected. Antibiotic annotation based on metadata revealed a correlation between antibiotic detection and microbial food metabolism. We observed the breakdown of soyasaponin across animals (Fig. 2d, ranges of other animals indicated by light gray shading) and a stark absence of the aglycone, soyasapogenol C, when antibiotics were empirically detected in the feces by MS (Fig. 2d to f).

Fecal samples from Isoka (Rx antibiotic) have a predominance of soyasaponin I (Fig. 2d). A longitudinal representation of the relative abundance of these two features for Isoka (Fig. 2d and e, black and dark blue) showed an initial ability to metabolize soyasaponin I to soyasapogenol C, which virtually disappeared upon commencing antibiotic treatment. In agreement with the MS-based detection of antibiotics in multiple fecal samples from Okubi (Fig. 2f), we also observed a diminished ability to metabolize soyasaponin, which has a stochastic nature, similar to the intermittent observation of antibiotics (Fig. 2d and e, light gray and light blue shading). The remaining cheetahs have higher relative concentrations of soyasapogenol C (Fig. 2e, ranges indicated by light blue shading), indicating active microbial deglycosylation.

### Empirically derived metadata categorization avoids misinterpretation.

Due to the empirical evidence, a new metadata variable was created to delineate the difference between reported and detected antibiotic use, enabling metabolome-informed analyses.

**FIG 3:**
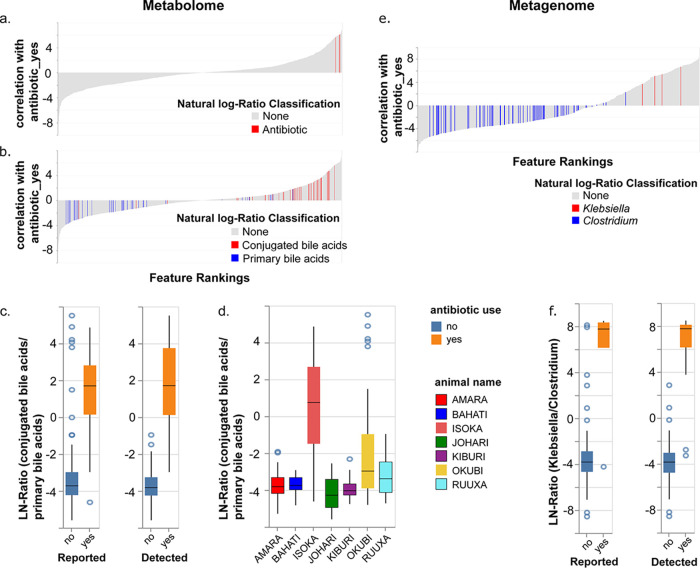
Metabolome data-informed groupings reveal impacts on the metabolome and microbiome profiles. (a and b) Differential abundance analysis of MS features (association with antibiotic_yes compared to antibiotic_no as the reference) visualized with Qurro. Positive values in the rank plot correspond to a positive association with antibiotic use. (a) Antibiotic features highlighted in red. (b) Impact of antibiotics on bile acid metabolism. Conjugated bile acids are shown in red, and primary bile acids are shown in blue. (c and d) Natural log ratio of conjugated bile acid features by primary bile acid features for reported versus detected antibiotic metadata categorization (c) and plotted by animal (d). (e) Differential abundance analysis of MS features (association with antibiotic_yes compared to antibiotic_no as the reference) visualized with Qurro. Positive values in the rank plot correspond to a positive association with antibiotic use. Numerator: *Klebsiella* genome IDs (red); Denominator: *Clostridium* genome IDs (blue). (f) Natural log ratio of *Klebsiella* to *Clostridium* for reported versus detected antibiotic metadata sorting, respectively. Note the removal of outliers in “reported – no” and “detected – no”. The difference between antibiotic use and no antibiotic use for reported and detected in panels c and f are statistically significant (*P* < 1e−31 for all), based on a Welch’s *t* test.

**(i) Metabolome-informed metabolome analysis.** Antibiotics imparted a strong signature to the differences between samples, both in terms of the medication itself, metabolism of soy carbohydrates, as discussed above, as well as changes in host and host-microbial metabolism. The hydrolyzed amoxicillin detected by MS was positively correlated with antibiotic use and highly ranked (Fig. 3a), based on differential abundance analysis. Furthermore, antibiotic use had a profound influence on bile acid metabolism. Conjugated bile acids correlate with antibiotic use (Fig. 3b), whereas primary bile acids are less likely to be observed after antibiotic exposure. The natural log ratios of conjugated (primary and secondary) bile acids divided by primary bile acids were elevated for Isoka and Okubi (Fig. 3d), and the difference between the MS-metadata informed categories can be clearly seen in Fig. 3c, left and right, respectively. In both cases, the difference between antibiotic use and not is statistically significant (Welch’s *t* test, *P* values of 4.5e−49 [MS_reported] and 1.3e−31 [MS_detected]). In particular, several apparent outliers in the “no antibiotic use reported” group are no longer outliers, as they are positioned within the newly corrected metadata category “antibiotic-detected.”

Furthermore, application of this informed metadata variable to the metabolite data still showed that the main driver in separation of cheetah fecal material was antibiotic detection, with PC1 representing 32.37% of the variance in the data set and effectively separating Isoka and some of Okubi’s samples from the remaining animals (see Fig. S1a in the supplemental material). Based on a permutational multivariate analysis of variance (PERMANOVA) analysis of the weighted UniFrac distance matrix, categorization based on metabolite data resulted in a larger effect size (*F* statistic for the two different metadata categories, antibiotic use [reported] = 28.2433 compared with antibiotic_presence_WD [detected] = 47.4572.).

10.1128/mSystems.00635-19.3 FIG S1 Antibiotics are clearly differentiated in the metabolome (a) and metagenome (b) when using MS-informed metadata grouping. Principal-coordinate analysis (weighted UniFrac) for metabolome (left) and metagenome (right) for fecal samples from seven cheetahs housed at Wildlife Discoveries. Data points are colored by MS-informed detected antibiotic use based on empirical evidence (blue for no; red for yes). The shape of the symbol designates the disease state, with regards to cheetah liver necrosis syndrome (CLNS). Download FIG S1, TIF file, 1.9 MB. Copyright © 2020 Gauglitz et al. 2020 Gauglitz et al. This content is distributed under the terms of the Creative Commons Attribution 4.0 International license.

Thus, one loop of application of the MS-informed metadata has been completed, applying a new categorization of antibiotic use that reflects the mass spectral data. Recategorization will impact data interpretation: removal of these samples from further analysis reduces the chances of data misinterpretation due to the outliers originally present. Furthermore, the same changes applied based on the MS-informed metadata can be applied to any additional paired data set.

**(ii) Metabolome-informed metagenome analysis.** The metagenome results also have a larger effect size for the metadata assigned due to metabolite analysis (detected) compared with the original metadata (reported) (PERMANOVA *F* statistic calculated from the weighted UniFrac distance matrix, 41.7599 for antibiotic_use [reported] compared with 75.9906 for antibiotic_presence_WD [detected]) (Fig. 1b versus Fig. S1b). The same trend held true for the unweighted distance matrix (Fig. S2): *F* statistic of 79.4844 for antibiotic_use (reported) compared with 139.961 for antibiotic_presence_WD (detected). Removal of the antibiotic use samples prior to PCoA results in removal of the strong antibiotic signature and reveals a new distribution of samples (Fig. S3). Many of Johari’s samples (severe cheetah liver necrosis syndrome [CLNS]) group together in the upper left corner, and samples for Amara, with mild CLNS, do not have distinct clustering from the remainder of the healthy fecal samples.

10.1128/mSystems.00635-19.4 FIG S2 Antibiotics are also clearly differentiated in the metagenome when using MS-informed metadata grouping and unweighted UniFrac. Data points are colored by MS-informed detected antibiotic use based on empirical evidence (blue for no; red for yes). The shape of the symbol designates the disease state, with regards to cheetah liver necrosis syndrome (CLNS). Download FIG S2, TIF file, 1.5 MB. Copyright © 2020 Gauglitz et al. 2020 Gauglitz et al. This content is distributed under the terms of the Creative Commons Attribution 4.0 International license.

10.1128/mSystems.00635-19.5 FIG S3 Principal-coordinate analysis (weighted UniFrac) for shotgun sequence data for WD animals, excluding Isoka and Okubi, colored by individual. The shape of the symbol designates the disease state, with regards to cheetah liver necrosis syndrome (CLNS). Download FIG S3, TIF file, 1.4 MB. Copyright © 2020 Gauglitz et al. 2020 Gauglitz et al. This content is distributed under the terms of the Creative Commons Attribution 4.0 International license.

The representation of taxa within metagenomic samples from antibiotic-treated animals is likely to be different than that from healthy controls. We assessed the difference in the taxonomic composition of samples without antibiotic use reported initially (reported) and then with the addition of samples in which antibiotics were detected (detected) (as determined by mass spectral data). There is a stark impact of antibiotic use on the microbial composition of the cheetah fecal samples on antibiotic use (Fig. S4, top versus bottom), which is further differentiated when applying the MS-detected antibiotic grouping (Fig. S4, right-hand side). Notably, *Klebsiella* is no longer observed among the top 10 most abundant genera when the samples are grouped by antibiotic detection rather than by reporting. *Klebsiella* is also positively associated with antibiotic use, while genera such as *Clostridium* are negatively correlated with antibiotic use (Fig. 3e). The natural log ratio of *Klebsiella* to *Clostridium* for antibiotic use is higher for antibiotic use in both reported and detected metadata categories (Welch’s *t* test, *P* value 7.7e−61 [detected] and 6.5e−80 [reported]); however, notable outliers with a high ratio in antibiotic reported samples in the “no” category are correctly categorized with the application of the metabolome-informed metadata categorization (Fig. 3f).

10.1128/mSystems.00635-19.6 FIG S4 Taxon bar plots from shotgun sequence data showing differences in antibiotic partitioning with and without metabolome data. (Left) Top 10 genera observed in samples grouped based on the original reported metadata. (Right) Top 10 genera observed in samples grouped based on mass spectral analysis (not detected). *Klebsiella* abundance in the samples with no antibiotic use decreases from reported (4.8%) to empirically detected (0.27%). The remaining taxa are included in the “Other” category. Download FIG S4, TIF file, 1 MB. Copyright © 2020 Gauglitz et al. 2020 Gauglitz et al. This content is distributed under the terms of the Creative Commons Attribution 4.0 International license.

### MS-informed refinement of feature table.

**FIG 4:**
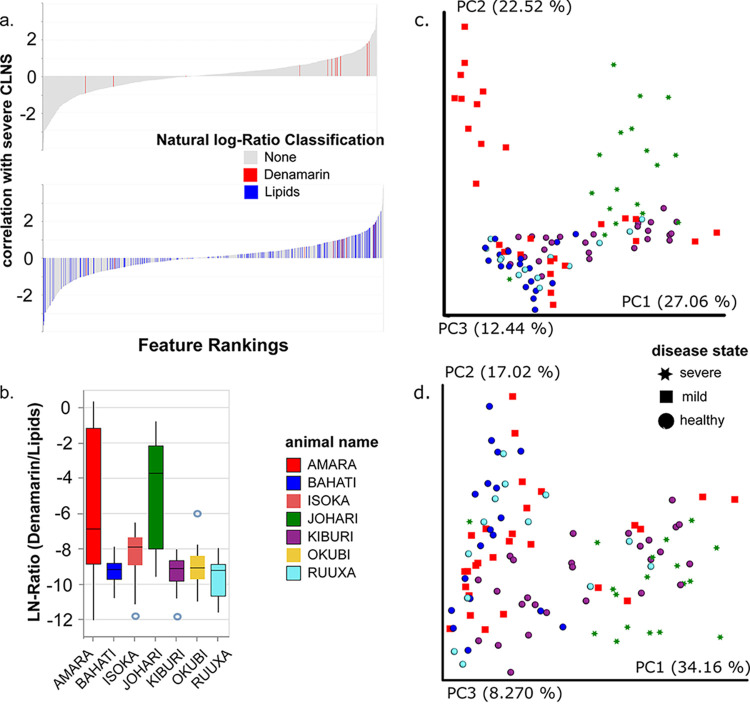
Metabolome-informed filtering of metabolite feature table to remove medication metabolites. Differential abundance analysis of MS features (association with severe liver disease compared to healthy as the reference) visualized with Qurro. Positive values in the rank plots correspond to a positive association with liver disease. (a, top) Denamarin features highlighted in red; (bottom) denamarin features in red and lipid features in blue as a reference. (b) Natural log ratio of denamarin features by the general category lipids plotted by animal. Amara and Johari, both diseased animals, have increased levels of denamarin compared to the other animals. (c and d) A PCoA analysis (weighted UniFrac) of 103 samples from WD (five animals, all with no antibiotic exposure), with all features (c) show a distinct separation for Amara (red) and Johari (green), while a PCoA analysis (weighted UniFrac) with the features from the metabolism of the supplement denamarin removed (d) shows this difference along PC2 was due to this confounder. (Denamarin is defined as the following metabolites: adenine, 5′-methylthioadenosine, *S*-(5′-adenosyl)-l-methionine cation). In panel d, Johari separates along PC1 from healthy animals, but with some overlap from Kiburi and Amara. The shape of the symbol designates the disease state, with regard to cheetah liver necrosis syndrome (CLNS). All values are colored by the source (individual cheetahs).

Cases of metadata inaccuracy due to subjective reporting, missing metadata, or unexplained observations have been noted in the literature (8), as well as in this article. Regardless of the origin of such observations, it is important to develop strategies to mitigate their impact on the conclusions drawn with regard to the question of interest, and empirically derived information allows us to gain insights beyond the information initially collected. MS analysis revealed the presence of *S*-adenosyl methionine metabolites (from the supplement denamarin) present in the samples from the diseased cheetahs, Amara and Johari, the only ones given the supplement. These features are differentially abundant (Fig. 4a) and are positively correlated with disease compared with the healthy reference samples. These features, compared with a natural log ratio of a general class of all annotated lipids in the metabolite data set, spread broadly across the rankings (Fig. 4a, bottom), are more abundant in samples from Amara and Johari, compared to the remaining cheetahs, including those on antibiotics (Fig. 4b).

Metabolome data-informed grouping, which was empirically validated, could be used to stratify individuals, to identify criteria of interest, or to flag samples for exclusion from further analysis. In this case, removal of samples from animals exposed to antibiotics, Isoka and Okubi, and recomputation of the PCoA of metabolite data revealed new trends (Fig. 4c). Exclusion of samples from Isoka and Okubi (Fig. 4c) resulted in no per individual differences along PC1; however, there was a clear separation of Johari (severe disease) and Amara (mild disease) from the remaining healthy individuals along PC2, representing 22.52% of the variance.

We hypothesized that the strong signature from denamarin metabolites was driving this difference in beta diversity, and a further iteration of MS-informed analysis was performed (Fig. 1). The untargeted metabolomic feature table was filtered to remove metabolite signatures for *S*-adenosyl methionine, as well as its breakdown products, from the metabolite feature table (red in Fig. 4a). Analysis of the modified set of features resulted in decreased separation of metabolite signatures from the cheetahs Johari and Amara (Fig. 4d). We observed that along PC1, Johari, with severe CLNS (data points displayed as green stars), was most disparate, while Amara, with a diagnosed mild case (red squares) and improving clinical readouts (Table S2), was not clearly distinguishable from the healthy individuals, as seen in the metagenomic analysis as well.

10.1128/mSystems.00635-19.2 TABLE S2 Additional clinical measurements for diseased cheetahs Johari and Amara. Samples taken on 23 January 2018, highlighted in green, were collected during the sampling period. Those samples from Amara highlighted in yellow are referenced in the text. Expected Results (Based on Best Available Match): Type: Min-Max | Mean [Median] *N* (Animals). ALT: Global subsp RI: 30−185 | 93 [85] *N* = 361 (113 animals). AST: Global subsp RI: 22−91 | 47 [44] **N** = 334 (99 animals). The reference range for total bile acids is 0 to 6.9 μmol/liter. Moderate to severe elevation (greater than 30) is generally consistent with hepatic dysfunction. Download Table S2, CSV file, 0.01 MB. Copyright © 2020 Gauglitz et al. 2020 Gauglitz et al. This content is distributed under the terms of the Creative Commons Attribution 4.0 International license.

Although the sample size is small, as is common with studies of captive wildlife, a comparison of clinical data for Amara and Johari (Table S2) support the groupings observed in the final metabolome-informed analyses (Fig. 4). Johari had the most severe case of CLNS and had liver enzyme levels of alanine aminotransferase (ALT) of 451 μmol/liter and aspartate aminotransferase (AST) of 414 μmol/liter on 23 January 2018 (critically elevated above the mean reported healthy values ALT of 47 μmol/liter for ALT and of 93 μmol/liter for AST). Amara, who is not clearly distinguished from healthy individuals, had elevated ALT and AST levels of 273 and 108 μmol/liter in 2017, at the time of diagnosis by liver biopsy; however, on 20 March 2018, just after sampling for this study, her levels were within healthy limits at 61 and 13 μmol/liter for ALT and AST, respectively. Peripheral bile acid levels were similarly elevated for Johari as well as Amara in 2017 but within normal limits for Amara in 2018 (Table S2). The metabolome profile and presence of Amara’s samples with other healthy individuals is supported by the lower ALT, AST, and bile acid levels measured shortly after the sampling time period, indicating a possible recovery back to a healthier phenotype within this case study.

## DISCUSSION

There are confounders in clinical data sets that cannot be predicted based on available information. Broad untargeted metabolomics gives insights into specific molecules that can reveal unexpected factors that, if not understood, might lead to misinterpretation of the data. In this study, we observed an individual animal reported not to have been administered antibiotics during the study period but detected antibiotics in their stool. Taking this information into account to generate an MS-informed metadata category for antibiotic use allowed a more informative analysis of the samples. Similarly, upon observation of denamarin metabolites, their signatures could be removed as a result of treatment for liver disease, rather than attributing them as drivers of the condition.

In the current study, to explore the signatures of liver disease, the observation of unexpected antibiotics across numerous samples was very valuable; otherwise Okubi’s samples (no Rx) that contained antibiotics would continue to be included as “healthy,” which is true with respect to liver disease, but there is a substantial and clear shift in this cheetah’s microbiome due to antibiotic exposure. As antibiotics are a stronger driver of difference than the disease state, it was appropriate in this case to remove the samples from both animals from the analysis, as they would otherwise be classified as “healthy” because neither suffers from CLNS. We have thus demonstrated that MS-informed analyses driven by empirically detected information can reveal elusive information and go beyond even the best (and most intrusive) study information.

The applicability of our approach is most powerful in the case of restricted populations, as the detection of antibiotics in the stool of an “untreated” animal may have been facilitated by the contained environment in which these cheetahs live. Our approach has the potential to impact how the health of animals in captivity is monitored, across different social structures, as our method easily extends to uncovering and tracking intragroup metabolite transfer in other captive animal populations that engage in social grooming or shared living spaces, including domestic cats and dogs.

We observe that when two animals are housed in the same enclosure and one is treated with antibiotics, the effect of the antibiotic on the metabolome and metagenome is also seen in the cohoused individual. While this has not been observed in captive wildlife previously, it has been observed in humans in the case of the metagenome (e.g., reference 10). Uniquely, the antibiotic was also detected in the stool of the “untreated”’ animal. We cannot say for certain whether cohousing, grooming, or other social activities occurred in this instance; however, these results provide a testable hypothesis for managing antibiotic usage in captive wildlife.

Metabolomics generates a verifiable readout that allows us to reinterpret the study results in the context of the data or information that were actually collected and can be done as an iterative process. Regardless of the origin of such observations, whether it is metadata omission, misreporting, errors in sample collection, or other causes, the fact remains that something is detected where it was not expected. The power of using metabolomic data to reinterpret metadata categorization, impacting all facets of gut microbiome data interpretation, has a critical place in microbiome analyses going forward, and virtually any study with samples amenable to MS data collection could potentially benefit from this approach.

## MATERIALS AND METHODS

### Study design.

Between 2015 and 2018, multiple cheetahs from the San Diego Zoo Safari Park collection were diagnosed with cheetah liver necrosis syndrome (CLNS) via liver biopsy or postmortem exam. A working group, including clinical veterinarians, pathologists, nutritionists, and husbandry staff, met regularly to develop plans for earlier detection, treatment, and disease characterization and to elucidate the cause of the condition. Fecal samples were obtained from both sick and healthy cheetahs housed at Wildlife Discoveries (WD). Cheetahs included in this study are ambassador animals at the San Diego Zoo Safari Park, which means that they leave their enclosures regularly, and travel within the Park, where they interface with fomites in human and other wildlife spaces, and often leave the grounds. The project was exempt from IACUC review, as samples were collected noninvasively as part of a planned, potentially corrective, diet change. Fecal samples were shared between San Diego Zoo Safari Park and University of California, San Diego (UCSD) researchers following completion of an External Biomaterials Request in order to explore whether metabolomic and metagenomic data offered a more detailed biochemical picture of ingested materials, both dietary and environmental, beyond the limited nutritional analyses already available.

### Sample handling.

Fecal samples were collected each morning daily by trainers at WD for 4 weeks from mid-January 2018 to mid-February 2018. Fecal material was collected on location by animal care staff or transported to the clinical lab at Harter Veterinary Medical Center (HVMC) and sampled by a senior nutrition research associate. Samples were not subjected to extremes of heat or cold (i.e., direct sunlight, vehicle cab, or refrigerator/freezer) during transport. A fecal score was assigned to each fecal sample as follows: 1 for liquid/watery (runny), 2 for soft (loose, overly moist), 3 for formed (maintains shape), and 4 for hard/dry (firm, lacking moisture). Each sample was assigned a unique barcode identifier (ID), and a label was affixed to the swab tube and a small printed label was affixed to the 2-ml tube, making sure both samples had matching numbers for the same sample and animal.

Two separate samples were collected per individual cheetah, a swab of the fecal surface and a small amount of whole feces in a 2-ml microcentrifuge tube. The interior of the feces was swabbed with a sterile barcoded cotton swab (BD SWUBE applicator) when possible, or the exterior for smaller samples for sequence analysis. An aliquot of bulk stool was transferred to an empty 2-ml round bottom microcentrifuge tube for mass spectrometric analysis (Qiagen, Hilden, Germany). Samples were frozen at –80°C within 24 h of deposition at the San Diego Zoo Institute for Conservation Research (ICR). All samples were transported on dry ice and stored at –80°C until analysis.

All information regarding fecal samples for each animal were recorded into an Excel spreadsheet (Microsoft, Seattle, WA). Information included sample ID, location, date, animal name, institution ID, time feces deposited (if known), time feces collected, time feces sampled, fecal score, any medical/chronic problems (i.e., fecal quality, medications), and day and progression of diet transition.

### Metabolomic sample extraction.

All fecal samples were dried, weighed, and extracted to the same final concentration. Frozen cheetah fecal samples were placed on ice. The lid and rim of each sample tube were cleaned with a Kimwipes (Kimberly-Clark) moistened with 70% ethanol (EtOH) to remove spurious fecal material. Samples were dried overnight using a Labconco CentriVac and either placed at –80°C for storage or immediately processed as follows. A clean spatula and tweezers were used to transfer 50 to 100 mg of dried stool into a new, labeled tube. Exact weights were recorded, and a 10-fold volume of cold 50% methanol (MeOH) in water was added to each tube (for example, 50 mg of stool plus 500 μl of 50% MeOH) (liquid chromatography-mass spectrometry [LC-MS]-grade solvents; Fisher Chemical). The samples were homogenized for 5 min at 25 Hz on a tissue homogenizer (Qiagen TissueLyzer II; Qiagen, Hilden, Germany) and subsequently placed at –20°C for 15 min for methanol extraction. The samples were then centrifuged at maximum speed (14,000 × *g*) for 15 min (Eppendorf US centrifuge 5418; USA). Without disrupting the pellet, 300 μl of the supernatant was transferred into a 96-deep-well plate. Samples were sealed and stored at –80°C.

### Metabolomic data acquisition and processing.

Metabolomic data were collected using a modification of the data-dependent acquisition method outlined in reference 17. Briefly, extracts were dried down, resuspended in 50% MeOH−50% water (Optima LC-MS grade; Fisher Scientific, Fair Lawn, NJ, USA). Untargeted metabolomics was conducted using an ultrahigh-performance liquid chromatography system (UltiMate 3000; Thermo Fisher Scientific, Waltham, MA) coupled to a Maxis quadruple time of flight (Q-TOF) mass spectrometer (Bruker Daltonics, Bremen, Germany) with a Kinetex C_18_ column (Phenomenex, Torrance, CA, USA). A linear gradient was applied as follows: 0 to 0.5 min, isocratic at 5% mobile phase (MP) B; 0.5 to 8.5 min, 100% MP B; 8.5 to 11 min, isocratic at 100% MP B; 11 to 11.5 min, 5% MP B; 11.5 to 12 min, 5% MP B, where mobile phase A is water with 0.1% formic acid (vol/vol) and mobile phase B is acetonitrile−0.1% formic acid (vol/vol) (LC-MS grade solvents; Fisher Chemical). Electrospray ionization in the positive mode was used.

### MS1 feature finding and data processing.

qToF files (.d) were exported using DataAnalysis (Bruker) as .mzXML files after lock mass correction using hexakis (1H, 1H, 2H-difluoroethoxy) phosphazene (Synquest Laboratories, Alachua, FL), with *m/z* 622.029509. Data quality was assessed by qualitatively evaluating the *m/z* error and retention time of the LC-MS standard solution (i.e., mixture of six compounds), which was analyzed at least once in every 96-well plate.

MS1 feature finding was performed on the .mzXML files in MZmine2 (version MZmine-2.37.corr16.4) (18). The code for this version of mzMINE has been archived with the raw data files available on MassIVE under MSV000082969. The mzMINE parameters used for feature finding are as follows: mass detection (centroid; MS1, 1.5E3; MS2, 90); ADAP Chromatogram builder (minimum group size in number of scans, 4; group intensity threshold, 5E3; minimum highest intensity, 2E3; *m/z* tolerance, 0.001 *m/z* to 20 ppm); chromatogram deconvolution (local minimum search [LMS], chromatographic threshold of 96%, search minimum in retention time [RT] range [minutes] of 0.03, minimum relative height of 5%, minimum absolute height of 2E3, minimum ratio of peak top/edge of 1 and peak duration range [minutes] of 0 to 2; *m/z* center calculation set to auto; *m/z* range for MS2 scan pairing (daltons) of 0.02 and RT range for MS2 scan pairing (minutes) of 0.15); isotope peaks grouper (*m/z* tolerance set to 0.0015 *m/z* or 10 ppm; retention time tolerance of 0.05, maximum charge of 3; and representative isotope set to most intense); order peak lists; join aligner (*m/z* tolerance set at 0.0015 *m/z* or 15 ppm; weight for *m/z* of 2; retention time tolerance of 0.2 min; weight for RT of 1. A filter was used such that only features present in at least two samples were included. The output was a data matrix of variables (i.e., MS1 features that triggered MS2 scans) by samples, exported for global natural products (GNPS) (.mgf and .csv quant table) and for SIRIUS (.mgf). The MS1 data matrix (MS1 features from quant table) was processed by concatenating the *m/z* and retention time columns from the original MZmine output.

Feature-based molecular networking was performed, and library IDs were generated using GNPS (14). The quant table, SIRIUS export, and library identifications from feature-based molecular networking were used as inputs for the Qiime2 plug-in Qemistree (https://github.com/biocore/q2-qemistree) to perform hierarchical ordering of the untargeted mass spectrometry data. The resultant Qemistree-based feature table can be linked to the original feature number from feature finding (which also links to the GNPS library IDs) and presents fingerprints that act to merge spectra assigned the same identity. Qemistree also generates a fingerprint-based tree, allowing for tree-based approaches such as UniFrac (19, 20).

### Applicability of weighted UniFrac for Qemistree abundance analysis.

Mass spectrometry feature data represent relative abundances of compounds, not exact counts. Due to this difference, the use of unweighted methods, which look only for presence/absence, are not suitable. Unweighted UniFrac (20) would exaggerate differences between samples if even a small number of new molecules appears in one batch and not in another batch as frequently occurs due to instrument performance changes during sample processing. Parameters such as gap filling and feature finding parameter settings can further compound these issues. Therefore, weighted UniFrac (19) is used for all comparisons between mass spectral data which were processed using the QIIME 2 plug-in q2-qemistree.

QIIME 2 (21) was used within a jupyter notebook environment for principal-coordinate analysis. Differential abundance analysis was performed in command line using Songbird (https://github.com/biocore/songbird) (22) and visualized using Qurro (https://github.com/biocore/qurro).

### Sample preparation and sequencing data generation.

Shallow shotgun sequencing was performed as previously described (23). In brief, DNA extraction was performed using the Qiagen PowerSoil DNA extraction kit following the Earth Microbiome Project (EMP) standard protocol (24). The Qubit double-stranded DNA (dsDNA) high-sensitivity (HS) assay (Thermo Fisher Scientific) was used to determine concentration, and libraries were prepared from 1 ng of input DNA in a miniaturized Kapa HyperPlus protocol. Libraries were quantified using the Kapa Illumina library quantification kit, pooled, and size selected (300 to 800 bp) using the Sage Science PippinHT. The pooled library was sequenced as a paired-end 150-cycle run on an Illumina HiSeq 4000 system at the UCSD Institute for Genomic Medicine (IGM) Genomics Center. Demultiplexed sequences were trimmed and quality filtered using Atropos v 1.1.5, a fork of Cutadapt (25).

### Sequence data processing.

Significant host contamination was expected from the horse-, beef-, and rabbit-based diet of the cheetahs, as well as host DNA present in the samples. Therefore, we identified reads in the quality-filtered reads using Bowtie 2 v2.3.0 (26) with the “very-sensitive” parameter setting against the genomes of Acinonyx jubatus (cheetah, isolate AJU 981 Chewbacca, GCF_001443585.1), Bos taurus (cattle, Hereford breed, GCF_000003055.6), Equus caballus (horse, thoroughbred, isolate Twilight, GCF_002863925.1), and Oryctolagus cuniculus (rabbit, Thorbecke inbred, GCF_000003625.3) sequentially.

### Taxonomy generation and statistical analyses.

Host-filtered reads were mapped to the 10,575 genomes selected for phylogenetic reconstruction in the Web of Life project (https://biocore.github.io/wol/data/genomes/) (27) using Bowtie 2 within the alignment pipeline SHOGUN (12) using standard parameters. Bowtie 2 mappings were normalized to distribute reads to individual genomes, and the resulting output matrix was filtered to remove reads present at less than 0.01% relative abundance per sample. This filtered matrix was used for weighted UniFrac (19) and unweighted UniFrac (20) beta diversity analysis as well as taxonomic summarization using the Web of Life tree (https://biocore.github.io/wol/data/trees) with QIIME 2 (21). Visualizations were prepared using Emperor (28) and matplotlib (29). Permutational multivariate analysis of variance (PERMANOVA) (30) analysis was performed in QIIME 2 v. 2018.11 on metabolite and metagenomic distance matrices. The *F* statistic was reported as a measure of effect size. Jupyter notebooks of the analyses are available at http://github.com/knightlab-analyses.

Differential abundance of microbes and metabolites, individually, with regard to different metadata variables were calculated using Songbird (22).

The following formula was used to learn the differentials for the microbes

$$y_{\text{microbes},k}=\beta_{0}+\beta_{1}x_{\text{location},k}+\beta_{2}x_{\text{liver},k}$$

where *y*_microbes,_*_k_* is the vector of microbe abundances for a given sample *k* and *x*_location,_*_k_* is the location from which the sample was collected from. β_1_ is a vector corresponding to the microbe differentials with respect to the sampling location. This represents the log fold differential for each microbe between the sampling locations. *x*_liver,_*_k_* is an ordinal variable with four possible values for CLNS: severe, CLNS, mild, and healthy. β_2_ is a vector corresponding to the microbe differentials with respect to the liver health. β_0_ represents the intercept of the multinomial regression.

The same regression formula was used to learn the differentials for the metabolites.

$$y_{\text{metabolites}}=\beta_{0}+\beta_{1}x_{\text{location}}+\beta_{2}x_{\text{liver}}$$

Differentials were visualized in rank plots using Qurro (https://github.com/biocore/qurro), while microbe-metabolite cooccurrence probabilities were computed using mmvec (https://github.com/biocore/mmvec). These cooccurrence probabilities represent the probability of observing a metabolite given the microbe is observed. These conditional probabilities are estimated through a low-rank approximation with three principal axes.

### Data and code availability.

All data in this study are publicly available. Raw and processed shotgun sequencing data are available in Qiita (31) study 11872 (https://qiita.ucsd.edu/study/description/11872) and GNPS (14) using MassIVE (https://massive.ucsd.edu/) ID MSV000082969. The GNPS Networking job is available at https://gnps.ucsd.edu/ProteoSAFe/status.jsp?task=093798dffe2448239410c3d465ef9fea.

## References

[1] Cho I, Yamanishi S, Cox L, Methé BA, Zavadil J, Li K, Gao Z, Mahana D, Raju K, Teitler I, Li H, Alekseyenko AV, Blaser MJ. 2012. Antibiotics in early life alter the murine colonic microbiome and adiposity. Nature 488:621–626. doi:10.1038/nature11400. 22914093 PMC3553221

[2] Korpela K, Salonen A, Virta LJ, Kekkonen RA, Forslund K, Bork P, de Vos WM. 2016. Intestinal microbiome is related to lifetime antibiotic use in Finnish pre-school children. Nat Commun 7:10410. doi:10.1038/ncomms10410. 26811868 PMC4737757

[3] Jakobsson HE, Jernberg C, Andersson AF, Sjölund-Karlsson M, Jansson JK, Engstrand L. 2010. Short-term antibiotic treatment has differing long-term impacts on the human throat and gut microbiome. PLoS One 5:e9836. doi:10.1371/journal.pone.0009836. 20352091 PMC2844414

[4] Dethlefsen L, Relman DA. 2011. Incomplete recovery and individualized responses of the human distal gut microbiota to repeated antibiotic perturbation. Proc Natl Acad Sci U S A 108(Suppl 1):4554–4561. doi:10.1073/pnas.1000087107. 20847294 PMC3063582

[5] Vázquez-Baeza Y, Callewaert C, Debelius J, Hyde E, Marotz C, Morton JT, Swafford A, Vrbanac A, Dorrestein PC, Knight R. 2018. Impacts of the human gut microbiome on therapeutics. Annu Rev Pharmacol Toxicol 58:253–270. doi:10.1146/annurev-pharmtox-042017-031849. 28968189

[6] Johnson AJ, Vangay P, Al-Ghalith GA, Hillmann BM, Ward TL, Shields-Cutler RR, Kim AD, Shmagel AK, Syed AN, Personalized Microbiome Class Students, Walter J, Menon R, Koecher K, Knights D. 2019. Daily sampling reveals personalized diet-microbiome associations in humans. Cell Host Microbe 25:789–802.e5. doi:10.1016/j.chom.2019.05.005. 31194939

[7] David LA, Maurice CF, Carmody RN, Gootenberg DB, Button JE, Wolfe BE, Ling AV, Sloan Devlin A, Varma Y, Fischbach MA, Biddinger SB, Dutton RJ, Turnbaugh PJ. 2014. Diet rapidly and reproducibly alters the human gut microbiome. Nature 505:559–563. doi:10.1038/nature12820. 24336217 PMC3957428

[8] McDonald D, Hyde E, Debelius JW, Morton JT, Gonzalez A, Ackermann G, Aksenov AA, Behsaz B, Brennan C, Chen Y, DeRight Goldasich L, Dorrestein PC, Dunn RR, Fahimipour AK, Gaffney J, Gilbert JA, Gogul G, Green JL, Hugenholtz P, Humphrey G, Huttenhower C, Jackson MA, Janssen S, Jeste DV, Jiang L, Kelley ST, Knights D, Kosciolek T, Ladau J, Leach J, Marotz C, Meleshko D, Melnik AV, Metcalf JL, Mohimani H, Montassier E, Navas-Molina J, Nguyen TT, Peddada S, Pevzner P, Pollard KS, Rahnavard G, Robbins-Pianka A, Sangwan N, Shorenstein J, Smarr L, Song SJ, Spector T, Swafford AD, Thackray VG, Thompson LR, Tripathi A, Vázquez-Baeza Y, Vrbanac A, Wischmeyer P, Wolfe E, Zhu Q, American Gut Consortium, Knight R. 2018. American Gut: an open platform for citizen science microbiome research. mSystems 3:e00031-18. doi:10.1128/mSystems.00031-18. 29795809 PMC5954204

[9] Althubaiti A. 2016. Information bias in health research: definition, pitfalls, and adjustment methods. J Multidiscip Healthc 9:211–217. doi:10.2147/JMDH.S104807. 27217764 PMC4862344

[10] Abeles SR, Jones MB, Santiago-Rodriguez TM, Ly M, Klitgord N, Yooseph S, Nelson KE, Pride DT. 2016. Microbial diversity in individuals and their household contacts following typical antibiotic courses. Microbiome 4:39. doi:10.1186/s40168-016-0187-9. 27473422 PMC4967329

[11] Munson L, Terio KA, Worley M, Jago M, Bagot-Smith A, Marker L. 2005. Extrinsic factors significantly affect patterns of disease in free-ranging and captive cheetah (Acinonyx jubatus) populations. J Wildl Dis 41:542–548. doi:10.7589/0090-3558-41.3.542. 16244064

[12] Hillmann B, Al-Ghalith GA, Shields-Cutler RR, Zhu Q, Gohl DM, Beckman KB, Knight R, Knights D. 2018. Evaluating the information content of shallow shotgun metagenomics. mSystems 3:e00069-18. doi:10.1128/mSystems.00069-18. 30443602 PMC6234283

[13] Høiby N, Pers C, Johansen HK, Hansen H, The Copenhagen Study Group on Antibiotics in Sweat. 2000. Excretion of beta-lactam antibiotics in sweat—a neglected mechanism for development of antibiotic resistance? Antimicrob Agents Chemother 44:2855–2857. doi:10.1128/AAC.44.10.2855-2857.2000. 10991872 PMC90163

[14] Wang M, Carver JJ, Phelan VV, Sanchez LM, Garg N, Peng Y, Nguyen DD, Watrous J, Kapono CA, Luzzatto-Knaan T, Porto C, Bouslimani A, Melnik AV, Meehan MJ, Liu W-T, Crüsemann M, Boudreau PD, Esquenazi E, Sandoval-Calderón M, Kersten RD, Pace LA, Quinn RA, Duncan KR, Hsu C-C, Floros DJ, Gavilan RG, Kleigrewe K, Northen T, Dutton RJ, Parrot D, Carlson EE, Aigle B, Michelsen CF, Jelsbak L, Sohlenkamp C, Pevzner P, Edlund A, McLean J, Piel J, Murphy BT, Gerwick L, Liaw C-C, Yang Y-L, Humpf H-U, Maansson M, Keyzers RA, Sims AC, Johnson AR, Sidebottom AM, Sedio BE, Klitgaard A, . 2016. Sharing and community curation of mass spectrometry data with Global Natural Products Social Molecular Networking. Nat Biotechnol 34:828–837. doi:10.1038/nbt.3597. 27504778 PMC5321674

[15] Sumner LW, Amberg A, Barrett D, Beale MH, Beger R, Daykin CA, Fan T-M, Fiehn O, Goodacre R, Griffin JL, Hankemeier T, Hardy N, Harnly J, Higashi R, Kopka J, Lane AN, Lindon JC, Marriott P, Nicholls AW, Reily MD, Thaden JJ, Viant MR. 2007. Proposed minimum reporting standards for chemical analysis Chemical Analysis Working Group (CAWG) Metabolomics Standards Initiative (MSI). Metabolomics 3:211–221. doi:10.1007/s11306-007-0082-2. 24039616 PMC3772505

[16] Hu J, Zheng YL, Hyde W, Hendrich S, Murphy PA. 2004. Human fecal metabolism of soyasaponin I. J Agric Food Chem 52:2689–2696. doi:10.1021/jf035290s. 15113177

[17] Gauglitz JM, Aceves CM, Aksenov AA, Aleti G, Almaliti J, Bouslimani A, Brown EA, Campeau A, Caraballo-Rodríguez AM, Chaar R, da Silva RR, Demko AM, Di Ottavio F, Elijah E, Ernst M, Ferguson LP, Holmes X, Jarmusch AK, Jiang L, Kang KB, Koester I, Kwan B, Li J, Li Y, Melnik AV, Molina-Santiago C, Ni B, Oom AL, Panitchpakdi MW, Petras D, Quinn R, Sikora N, Spengler K, Teke B, Tripathi A, Ul-Hasan S, van der Hooft JJJ, Vargas F, Vrbanac A, Vu AQ, Wang SC, Weldon K, Wilson K, Wozniak JM, Yoon M, Bandeira N, Dorrestein PC. 2020. Untargeted mass spectrometry-based metabolomics approach unveils molecular changes in raw and processed foods and beverages. Food Chem 302:125290. doi:10.1016/j.foodchem.2019.125290. 31404873

[18] Pluskal T, Castillo S, Villar-Briones A, Oresic M. 2010. MZmine 2: modular framework for processing, visualizing, and analyzing mass spectrometry-based molecular profile data. BMC Bioinformatics 11:395. doi:10.1186/1471-2105-11-395. 20650010 PMC2918584

[19] Lozupone CA, Hamady M, Kelley ST, Knight R. 2007. Quantitative and qualitative β diversity measures lead to different insights into factors that structure microbial communities. Appl Environ Microbiol 73:1576–1585. doi:10.1128/AEM.01996-06. 17220268 PMC1828774

[20] Lozupone C, Knight R. 2005. UniFrac: a new phylogenetic method for comparing microbial communities. Appl Environ Microbiol 71:8228–8235. doi:10.1128/AEM.71.12.8228-8235.2005. 16332807 PMC1317376

[21] Bolyen E, Rideout JR, Dillon MR, Bokulich NA, Abnet CC, Al-Ghalith GA, Alexander H, Alm EJ, Arumugam M, Asnicar F, Bai Y, Bisanz JE, Bittinger K, Brejnrod A, Brislawn CJ, Brown CT, Callahan BJ, Caraballo-Rodríguez AM, Chase J, Cope EK, Da Silva R, Diener C, Dorrestein PC, Douglas GM, Durall DM, Duvallet C, Edwardson CF, Ernst M, Estaki M, Fouquier J, Gauglitz JM, Gibbons SM, Gibson DL, Gonzalez A, Gorlick K, Guo J, Hillmann B, Holmes S, Holste H, Huttenhower C, Huttley GA, Janssen S, Jarmusch AK, Jiang L, Kaehler BD, Kang KB, Keefe CR, Keim P, Kelley ST, Knights D, Koester I, . 2019. Reproducible, interactive, scalable and extensible microbiome data science using QIIME 2. Nat Biotechnol 37:852–857. doi:10.1038/s41587-019-0209-9. 31341288 PMC7015180

[22] Morton JT, Marotz C, Washburne A, Silverman J, Zaramela LS, Edlund A, Zengler K, Knight R. 2019. Establishing microbial composition measurement standards with reference frames. Nat Commun 10:2719. doi:10.1038/s41467-019-10656-5. 31222023 PMC6586903

[23] Marotz CA, Sanders JG, Zuniga C, Zaramela LS, Knight R, Zengler K. 2018. Improving saliva shotgun metagenomics by chemical host DNA depletion. Microbiome 6:42. doi:10.1186/s40168-018-0426-3. 29482639 PMC5827986

[24] Thompson LR, Sanders JG, McDonald D, Amir A, Ladau J, Locey KJ, Prill RJ, Tripathi A, Gibbons SM, Ackermann G, Navas-Molina JA, Janssen S, Kopylova E, Vázquez-Baeza Y, González A, Morton JT, Mirarab S, Zech Xu Z, Jiang L, Haroon MF, Kanbar J, Zhu Q, Jin Song S, Kosciolek T, Bokulich NA, Lefler J, Brislawn CJ, Humphrey G, Owens SM, Hampton-Marcell J, Berg-Lyons D, McKenzie V, Fierer N, Fuhrman JA, Clauset A, Stevens RL, Shade A, Pollard KS, Goodwin KD, Jansson JK, Gilbert JA, Knight R, Earth Microbiome Project Consortium. 2017. A communal catalogue reveals Earth’s multiscale microbial diversity. Nature 551:457–463. doi:10.1038/nature24621. 29088705 PMC6192678

[25] Didion JP, Martin M, Collins FS. 2017. Atropos: specific, sensitive, and speedy trimming of sequencing reads. PeerJ 5:e3720. doi:10.7717/peerj.3720. 28875074 PMC5581536

[26] Langmead B, Salzberg SL. 2012. Fast gapped-read alignment with Bowtie 2. Nat Methods 9:357–359. doi:10.1038/nmeth.1923. 22388286 PMC3322381

[27] Zhu Q, Mai U, Pfeiffer W, Janssen S, Asnicar F, Sanders JG, Belda-Ferre P, Al-Ghalith GA, Kopylova E, McDonald D, Kosciolek T, Yin JB, Huang S, Salam N, Jiao J-Y, Wu Z, Xu ZZ, Cantrell K, Yang Y, Sayyari E, Rabiee M, Morton JT, Podell S, Knights D, Li W-J, Huttenhower C, Segata N, Smarr L, Mirarab S, Knight R. 2019. Phylogenomics of 10,575 genomes reveals evolutionary proximity between domains Bacteria and Archaea. Nat Commun 10:5477. doi:10.1038/s41467-019-13443-4. 31792218 PMC6889312

[28] Vázquez-Baeza Y, Pirrung M, Gonzalez A, Knight R. 2013. EMPeror: a tool for visualizing high-throughput microbial community data. Gigascience 2:16. doi:10.1186/2047-217X-2-16. 24280061 PMC4076506

[29] Hunter JD. 2007. Matplotlib: a 2D graphics environment. Comput Sci Eng 9:90–95. doi:10.1109/MCSE.2007.55.

[30] Anderson MJ. 2001. A new method for non-parametric multivariate analysis of variance. Austral Ecol 26:32–46. doi:10.1046/j.1442-9993.2001.01070.x.

[31] Gonzalez A, Navas-Molina JA, Kosciolek T, McDonald D, Vázquez-Baeza Y, Ackermann G, DeReus J, Janssen S, Swafford AD, Orchanian SB, Sanders JG, Shorenstein J, Holste H, Petrus S, Robbins-Pianka A, Brislawn CJ, Wang M, Rideout JR, Bolyen E, Dillon M, Caporaso JG, Dorrestein PC, Knight R. 2018. Qiita: rapid, web-enabled microbiome meta-analysis. Nat Methods 15:796–798. doi:10.1038/s41592-018-0141-9. 30275573 PMC6235622

