# Supplementary figures and images for: Metabolome-Informed Microbiome Analysis Refines Metadata Classifications and Reveals Unexpected Medication Transfer in Captive Cheetahs
Source: mSystems. 2020 Mar 10;5(2):e00635-19. doi: 10.1128/mSystems.00635-19 (PMC7065514; doi:10.1128/mSystems.00635-19)

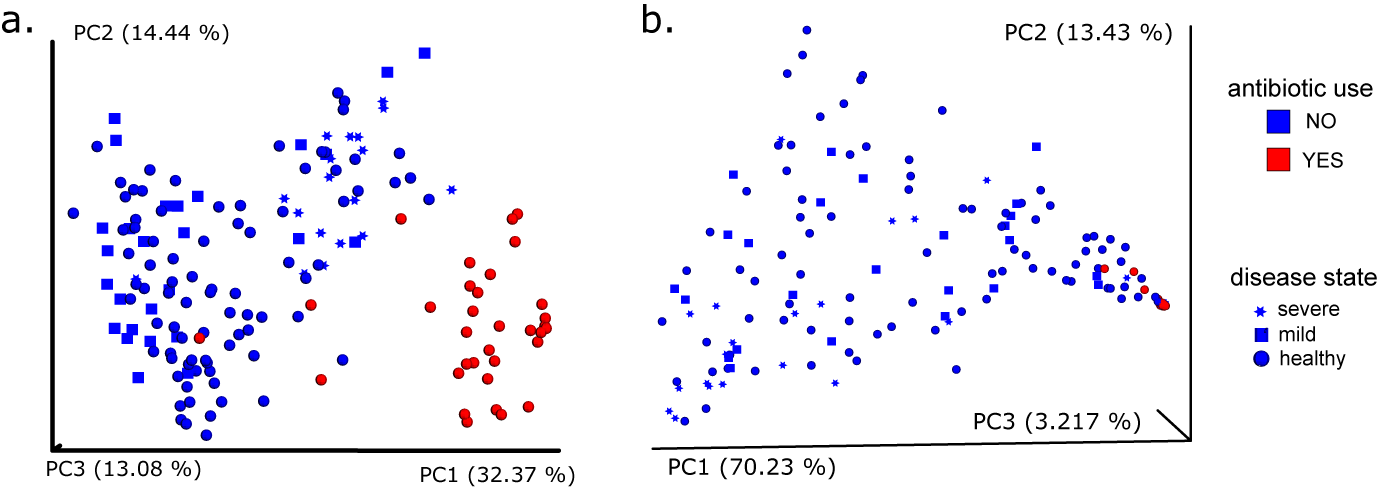

Supplement: FIG S1 [file mSystems.00635-19-sf001.tif]

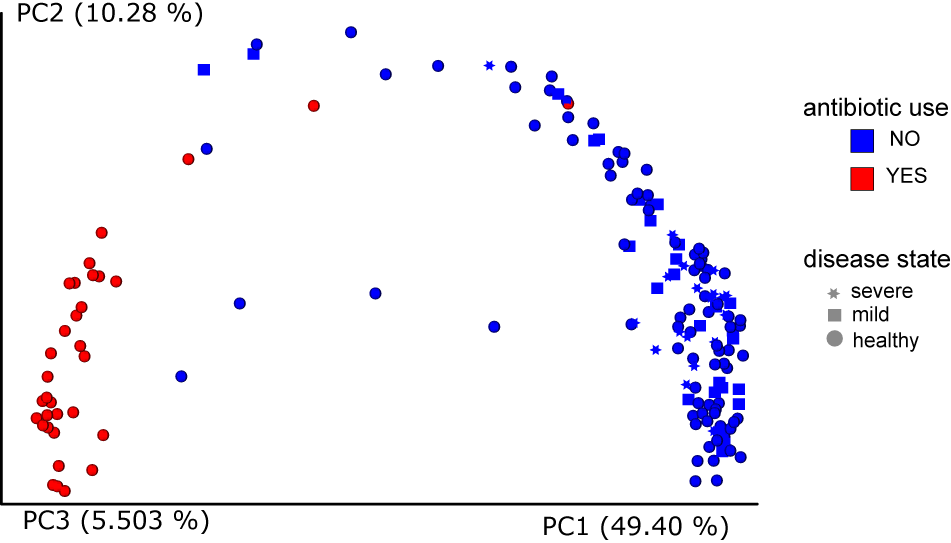

Supplement: FIG S2 [file mSystems.00635-19-sf002.tif]

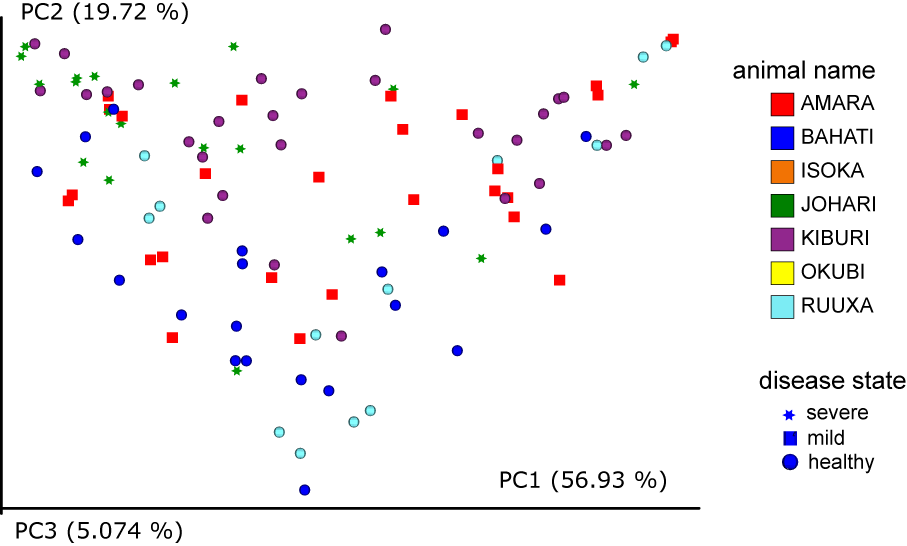

Supplement: FIG S3 [file mSystems.00635-19-sf003.tif]

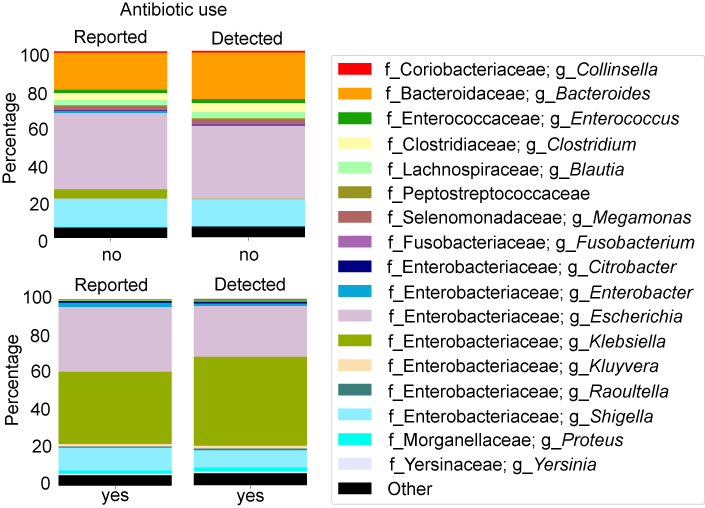

Supplement: FIG S4 [file mSystems.00635-19-sf004.tif]
